# Concentration and retention of chlorophyll around the extrafloral nectary of *Mallotus japonicus*

**DOI:** 10.1002/ece3.2959

**Published:** 2017-04-25

**Authors:** Akira Yamawo, Nobuhiko Suzuki

**Affiliations:** ^1^ Department of BiologyFaculty of Agriculture and Life ScienceHirosaki UniversityHirosakiJapan; ^2^ Department of Applied Biological SciencesFaculty of AgricultureSaga UniversitySagaJapan

**Keywords:** ant–plant mutualism, indirect defense, leaf age, resource allocation

## Abstract

Plants need to allocate some of their limited resources for defense against herbivores as well as for growth and reproduction. However, the priority of resource allocation within plants has not been investigated. We hypothesized that plants with extrafloral nectaries (EFNs) invest more chlorophyll around their EFNs—to support a high rate of carbon fixation there—than in other leaf parts of young leaves. Additionally, this chlorophyll may remain around EFNs rather than in the other leaf parts. We used *Mallotus japonicus* plants to investigate the chlorophyll content at leaf centers and edges and around EFNs at four stages of leaf development: middle‐expanded young leaves, fully expanded mature leaves, senior leaves, and leaves prior to abscission. These four stages of development were located at the third, fifth, eighth, and eleventh leaf positions from the apex, respectively. The results revealed that the chlorophyll content around the EFN side of the third‐position leaves was higher than that at the leaf center or edge. Although the chlorophyll content in the fifth‐position leaves did not differ between those at the leaf edge and around EFNs, the chlorophyll content around EFNs in the eighth‐position leaves was higher than that at the leaf centre and edge. The volume of EF nectar was positively correlated with the chlorophyll content around EFN during the leaf stage, but it was not correlated with the chlorophyll content in the leaf center and edge, except in fifth‐position leaves. These findings suggest that *M. japonicus* plants facilitate and maintain secretion of EF nectar in their young and old leaves, respectively, through the concentration and retention of chlorophyll around EFNs.

## 1 Introduction

Herbivores often strongly affect plant growth and reproduction. Therefore, plants need to allocate some of their limited resources to defend themselves against herbivores as well as to grow and reproduction (Walters, 2011; Yamawo, Tokuda, Katayama, Yahara, & Tagawa, 2015). Nevertheless, how resource allocation within plants is prioritized has not been investigated yet (but see Heil et al., 2002).

The optimal resource allocation to various plant defenses was proposed by McKey (1974, 1979) and this is now referred to as “optimal defense theory.” The optimal defense tactics are determined according to the value of the defended plant organs, physiological cost of the defense traits, and herbivore pressure; for example, leaf age affects the optimal defense tactics (van Dam, De Jong, Iwasa, & Kubo, 1996; Lambdon & Hassall, 2005; Yamawo, Suzuki, Tagawa, & Hada, 2012) because the leaf value, herbivore pressure, and physiological cost of particular defense traits change with the growth and ageing of leaves (Coley & Barone, 1996; Kielkiewicz & Van de Vrie, 1990; Meyer & Montgomery, 1987; Yamawo, Suzuki, et al., 2012).

New leaves of many plant species are often preferentially consumed by herbivores (Coley & Barone, 1996; van Dam, Verpoorte, & van der Meijden, 1994; McKey, 1974). Therefore, plants should be selected to pre‐emptively allocate their resources to defense traits. Trichomes can obstruct herbivore activity and they are often denser on young leaves obstructing herbivore activity, not only because young leaves have a high assimilative value and contain high concentrations of nutrients (Coley & Barone, 1996; Lambdon & Hassall, 2005; Radhika, Kost, Bartram, Heil, & Boland, 2008; Yamawo, Suzuki, et al., 2012) but also because they are not physically tough. New leaves must be soft; otherwise, the processes of rapid cell expansion and cell wall development cannot proceed. Several studies of fast‐growing plants have demonstrated that the chemical substances present in young leaves decrease with leaf age (van Dam et al., 1994; McKey, 1974; Yamawo, Suzuki, et al., 2012) as a result of the reallocation of these substances from maturing leaves to new leaves (van Dam et al., 1994; Radhika et al., 2008).

Furthermore, plants need to retain some defense traits in their old leaves. Plants are able to recycle internal nitrogen by resorbing nitrogen from senescing leaves (Yuan et al., 2005). This process enables plants to conserve and efficiently use nitrogen, a key nutrient that limits plant growth under many natural conditions (Chabot & Hicks, 1982; Chapin, 1980). Hence, defense traits present in old leaves may protect them to enable the resorption of nitrogen.

Extrafloral nectaries (EFNs) have been identified in approximately 4,000 species worldwide, including those in 745 genera from 108 families of flowering plants (Weber & Keeler, 2013). Many studies have reported on the defensive function of EFNs against herbivores (Koptur, 1992; Rico‐Gray & Oliveira, 2007). EFNs are plant glands that secrete sugar, water, and amino acids (Heil et al., 2000; Ness, 2003; Yamawo et al., 2015). Extrafloral (EF) nectar production depends on carbon fixation by the plant (Xu & Chen, 2015), which depends on the chlorophyll content of its leaves (Yamawo & Hada, 2010). We hypothesized that EFN‐bearing plants preferentially invest chlorophyll near EFNs, so as to support a high level of carbon fixation for EF nectar production, rather than in the other leaf parts of young leaves. Additionally, as it is needed for the continued production of EF nectar, this chlorophyll should remain around the EFNs more than at the other leaf parts.

Here, we focused on the plant *Mallotus japonicus* (L.) Muell. Arg. (Euphorbiaceae). We grew *M. japonicus* seedlings and investigated their chlorophyll content at the leaf center, leaf edge, and around the EFNs in leaves of various ages. We then discuss the resource allocation and resorption patterns.

## 2 Materials and Methods

### 2.1 Study species

We used seedlings of *M. japonicus*, a pioneer plant that grows in gaps and disturbed areas in temperate and subtropical regions of eastern Asia. The plant bears EFNs on its leaf edges (Yamawo, Katayama, Suzuki, & Hada, 2012), which secrete nectar that contains primary sugars used in the production of leaves (Yamawo et al., 2015). Instead, of the physical and chemical defenses provided by trichomes and pellucid dots, which function on young leaves, the EFNs mostly function on middle‐aged leaves and attract ants, thereby reducing leaf damage by eliminating herbivores from the plant (Yamawo, Tagawa, Hada, & Suzuki, 2014; Yamawo, Suzuki, et al., 2012). EF nectar secretion in middle‐aged leaves depends on the chlorophyll content of the leaves (Yamawo & Hada, 2010).

### 2.2 Cultivation of young *Mallotus japonicus* plants

Approximately 50 seeds of *M. japonicus* were collected from 10 trees growing in Okayama, western Japan (33°41′N, 133°55′E), during September and October 2011. A plastic container (45 × 35 × 15 cm) was filled with wet soil to a depth of 10 cm. The collected seeds were then sown at a 1‐cm depth during April 2012. This container was kept in a growth chamber at 35°C under a 12L:12D photoperiod for 24 h because the *M. japonicus* seeds germinate after experiencing high temperatures (Washitani & Takenaka, 1987). The container was kept at 25°C under the same photoperiod for approximately 2 months and watered every other day.

On 6 May 2012, a total of 21 plants were transplanted into plastic pots (20 × 20 × 25 cm) containing 70% tuff loam and 30% humus. The pots were placed in a greenhouse at Saga University (33°24′N, 130°29′E) and cultivated for 70 days. All of these plants had approximately 11–12 leaves at the beginning of the experiment.

### 2.3 Estimation of the chlorophyll content and extrafloral nectar production

To estimate the change in the chlorophyll content of each leaf part as a function of leaf age, we chose leaves at four positions: the third, fifth, eighth, and eleventh position from the apex of each plant. The corresponding ontogeny stages of these leaves were as follows: middle‐expanded young, fully expanded mature, senior, and leaves prior to abscission (Figure 2). We used one leaf per stage per plant. For each leaf stage, we estimated the chlorophyll content of a 6‐mm^2^ leaf area from each of the three leaf parts: around the EFNs at the leaf edge, at the leaf edge but 1 cm away from the nectar, and at the centre of the leaf. To do this, we used a chlorophyll meter (SPAD‐502, Konica Minolta). It measures the transmittance of red (650 nm) and infra‐red (940 nm) radiation through the leaf, and it calculates a relative SPAD meter value that should “correspond to the amount of chlorophyll present in the leaf sample” (Minolta, 1989). One EFN was randomly selected from each leaf per plant. The chlorophyll content (C) was estimated by the meter values (M) measured using the SPAD‐502 in following equation: $C\;=\;10^{\left(M^{0.265}\right)}$ (Markwell, Osterman, & Mitchell, 1995). The photosynthetic rate could be assessed using the values obtained from the SPAD‐502 because this rate is significantly correlated with the leaf chlorophyll content (Emerson, 1929; Fleischer, 1935).

To measure the nectar volume secreted from each EFN sampled to estimate the chlorophyll contents, selected leaves of cultivated plants were washed with distilled water to remove any accumulated EF nectar from the EFNs. The leaf surface was dried with Kim‐towels (Jujo Kimberly, Tokyo, Japan). Nectar secreted within 24* *h was then collected from each EFN by using 0.5‐μl microcapillary tubes (Drummond Scientific Co., Broomall, PA, USA). The volume of EF nectar was estimated by multiplying the proportion of the length of the tube filled with EFN to the total length by 0.5 μl.

### 2.4 Data analysis

All data were analyzed in R version 2.15.1 software (R Development Core Team, 2012).

The chlorophyll contents were examined by a generalized linear mixed model (GLMM) with a Poisson distribution with a log‐link function, including the genotype (parent plant ID) as a random effect, and by a chi‐squared test because the distribution of the residuals significantly differed from the normal distribution. Leaf age, positions within the leaf and their associations were included as explanatory variables. When the interaction was significant, we analyzed the effects positions within leaf on the volume of EF nectar by GLMMs with a Poisson distribution with a log‐link function, including the genotype as a random effect, and by a chi‐squared test. The chlorophyll contents were compared by a Steel–Dwass test. The relations between volume of EF nectar secreted and the estimated chlorophyll content of each position within the leaf were analyzed by GLMMs with a Gaussian distribution, including the genotype as a random effect, and by an *F*‐test. The positions within the leaf were included as explanatory variables.

## 3 Results

**Figure 1:**
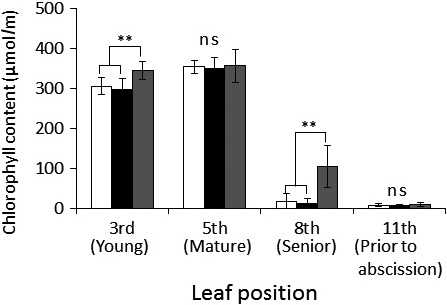
The estimated chlorophyll content at the leaf center, leaf edge, and around the extrafloral nectary at each leaf position. White column, leaf centre; black column, leaf edge; gray column, around extrafloral nectary (*n* = 21 for each column). The inside of bracket indicates the ontogenetic stage of each leaf position. Values are represented as mean ± SD **, significantly different at 1% level; ns, not significant (Steel–Dwass test)

The chlorophyll content differed among the positions within the leaves and leaf age classes (positions within leaves × leaf ages, GLMM, *df* = 6, χ^2^ **=* *18.87, *p *<* *.001). The chlorophyll content around the EFN side was higher than that at the leaf center and edge in the third‐ and eighth‐position leaves (Figure 1). On the contrary, the chlorophyll content did not differ between those at the leaf edges and those around EFNs in the fifth‐ and eleventh‐position leaves.

The third‐, fifth‐, eighth‐, and eleventh‐position leaves had the following EF nectar volumes: 0.02 ± 0.004 (mean ± SD), 0.02 ± 0.005, 0.003 ± 0.005, and 0.0003 ± 0.001μl, respectively. The volume of EF nectar was significantly and positively correlated with the chlorophyll content around the EFN during each leaf stage (Table 1), but did not correlate with the chlorophyll content in the leaf center and leaf edge, except in the mature leaves.

**Table 1:** Relations among the volume of extrafloral nectar (EFN) and estimated chlorophyll contents of leaf centre, leaf edge, and around the extrafloral nectary at each leaf position

Position within leaf	Leaf position								
	*df*	3rd		5th		8th		11th	
		*F*	Estimate	*F*	Estimate	*F*	Estimate	*F*	Estimate
Leaf edge	1	0.19	1.82	41.25	2.56^a^	0.34	2.48^a^	0.03	1.91
Leaf center	1	0.02	4.24	63.38	1.52^a^	0.79	−6.19^a^	0.31	−5.08
Around the EFN	1	9.80	1.16^a^	3.56	1.02^a^	12.63	5.66^a^	11.58	1.52^a^

a *p *< .01.

## 4 Discussion

In the present study, we found that *M. japonicus* plants have higher chlorophyll levels around the EFNs than in the other leaf parts of young leaves (Figure 1). Additionally, the chlorophyll remained around the EFNs until it was collected from other leaf parts prior to leaf senescence and abscission. One previous study has suggested the case for preferential investment in plant defense. Heil et al. (2002) also reported that *Macaranga triroba* invests more resources into increasing the production of food bodies and food for mutualist ants than into plant growth. Our findings may also suggest that *M. japonicus* plants preferentially invest their resources in defense.

With increasing leaf age, *M. japonicus* plants shift their anti‐herbivore defenses from physical and chemical traits to biotic using EFNs and food bodies (Yamawo, Suzuki, et al., 2012). This shift to biotic defense achieves a greater investment of resources for plant growth because the allocation costs of biotic defense are lower than those of physical and chemical defenses (Yamawo et al., 2015). The preferential investment of chlorophyll around EFNs may facilitate the ontogenic shift in leaf defenses and could be useful for minimizing the allocation cost of defense traits.

**Figure 2:**
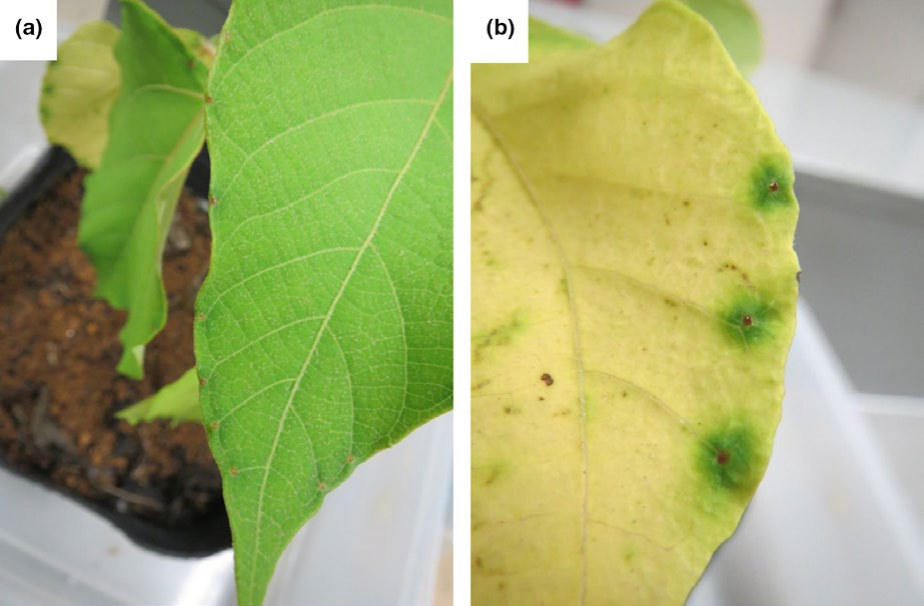
The extrafloral nectary of (a) third‐position and (b) eighth‐position leaves

The plants retained chlorophyll around the EFNs in the senior‐aged leaves (Figures 1 and 2). Yamawo, Suzuki, et al. (2012) demonstrated that EF nectar production was highest in the middle‐aged leaves, at approximately third to fifth positions from the shoot tip, but that it decreases with leaf age. However, prior studies have also demonstrated that leaves 7–11 weeks in age also secret a small amount of EF nectar which attracts some ants. Thus, the chlorophyll around EFNs in the senior leaves of *M. japonicus* may function to attract a small number of ant workers. Under such a condition, however, the defensive function against herbivory that is provided by ants on senior leaves appears to be small because its effectiveness depends on the abundance of ants attracted (Giusto, Anstett, Dounias, & McKey, 2001; Katayama & Suzuki, 2011; Yamawo, Hada, & Tagawa, 2017). Nonetheless, even a small number of ants might confer other beneficial functions to the plant, such as the exclusion of pathogens (Thornham, Smith, Ulmar Grafe, & Federle, 2012). If ant attraction in senior leaves has a defensive function against herbivores or pathogens, or both, it may increase the opportunity for the resorption of nitrogen from them, because it prevent consume these leaves and any nitrogen by herbivores or pathogen. Additional studies must be conducted to ascertain whether or not attracting ants to senior leaves is indeed beneficial for plants.

In conclusion, our results indicated the preferential concentration of chlorophyll around the EFNs over other parts in *M. japonicus* leaves during their development and functional maturity, to facilitate EF nectar production in young leaves. Furthermore, the plants retain the chlorophyll around the EFNs in the old leaves, and thus maintained the secretion of EF nectar. Together, these results support our hypothesis and provide a novel perspective for future studies on plant defense. Previous studies have focused on young leaves and plant tissues. Our results raise the intriguing question: “How does a plant defend old leaves containing resource elements”?

## Conflict of Interest

None declared.
